# Symptoms and coping of patients with dysphagia after anterior cervical spine surgery: a qualitative study

**DOI:** 10.1186/s12891-023-06621-5

**Published:** 2023-06-17

**Authors:** Chen Yu, Luo Chunmei, Song Caiping

**Affiliations:** 1 Department of Urology, Xinqiao Hospital, The Army Medical University, Chongqing, 400037 China; 2 Department of Orthopaedics, Xinqiao Hospital, The Army Medical University, Chongqing, 400037 China; 3 Department of Office of the hospital, Xinqiao Hospital, The Army Medical University, Chongqing, 400037 China

**Keywords:** Qualitative study, Anterior cervical spine surgery, Symptoms, Dysphagia

## Abstract

**Aim:**

To explore the subjective symptoms, psychological characteristics and coping strategies of patients with dysphagia after anterior cervical spine surgery, so as to provide the basis for formulating strategies to help patients with dysphagia solve clinical practice problems and to improve their quality of life after surgery.

**Methods:**

Using the phenomenological research method and the purpose sampling method, semi-structured interviews were conducted with 22 participants with dysphagia at 3-time points after anterior cervical spine surgery (7 days, 6 weeks, and 6 months).

**Results:**

A total of 22 (10 females and 12 males) patients, with years old ranging between 33 and 78 years were interviewed. When analysing the data, the following 3 categories were extracted from the participant interviews: “Subjective symptoms, Coping style and impact on social life”. The 3 categories consist of 10 sub-categories.

**Conclusions:**

The results showed that swallowing-related symptoms may occur after anterior cervical spine surgery. Many patients had developed compensatory strategies to manage or reduce the burden of these symptoms, but lacked professional guidance from health care professionals. Moreover, dysphagia after neck surgery has its unique characteristics, involving the interaction of physical, emotional and social factors, which requires early screening.Healthcare professionals should provide better psychological support in the early or late postoperative period to ensure the improvement of health outcomes and patients’ quality of life.

## Introduction

Anterior cervical spine surgery (ACSS) is a common operation,which has become the preferred treatment for cervical spondylosis due to it can directly and completely relieve the compression of the spinal cord or nerve root [1]. Many studies have reported that dysphagia is one of the most common complications after ACSS [2–4]. The reported incidence of dysphagia varies widely, probably due to the heterogeneity of existing literature [1]. In a well-designed prospective study, the incidence of postoperative dysphagia was as high as 71% [5].

Informed consent before anterior cervical surgery tends to focus on spinal cord compression, wound healing and potential complications [6]. The most common complications in this process are vocal cord paralysis and dysphagia [7]. Complaints may be discussed, but they are usually considered to be transient, so they are usually minimized in preoperative consultation. However, studies have found that ignoring early postoperative dysphagia can lead to more complications, such as sleep disorders, anorexia or asphyxia [8]. It even evolves into chronic dysphagia, which seriously affects the quality of life [9]. Therefore, we should pay attention to the prevention and management of dysphagia after ACSS.

At present, studies on dysphagia after ACSS mainly focus on the current situation and risk factors. Structured questionnaires will limit the answers of patients to a certain extent, so researchers should explore the views of patients as much as possible with open questions, so as to provide new insights into complex reality [10]. To our knowledge, no study has well described subjective dysphagia after ACSS from the perspective of patients, and no research has emphasized how it affects their quality of life and psychology. A rigorous qualitative approach is required to understand the subjective experience of dysphagia after ACSS. Therefore, through in-depth interviews, this study learned about the symptom experience and psychological feelings of dysphagia after ACSS and provided a reference for further improving the nursing management model of prevention of dysphagia after ACSS.

## Materials and methods

### Ethics considerations

Oral and written information about the study was distributed to each participating patient. We collected an informed and written consent form from each participant who did not object to the study. Patients who participated were told that they were free to accept or reject participation in the study and that this would not affect the quality of their care. The data were processed anonymously by a third party, who deleted the participant’s name and replaced them with a number. The qualitative data reported in this paper are from a clinical trial approved by the Ethics Review Committee of the Army Military Medical University, on June 22, 2022 (202,243,001).

### Type of study

The study was conducted between July to October 2022. It is a qualitative study based on interpretative phenomenology theory [11]. Using Colaizzi’s method for qualitative research, Semi-structured interviews provide a rich understanding of informants’ experiences of the world expressed in their own words. Colaizzi’s phenomenological approach focuses on the experiences and feelings of participants and finds common patterns in research objects rather than personal characteristics. This scientific method can ensure the authenticity of the experience gathered by participants in compliance with scientific standards.

### Patient Population and inclusion criteria

A purposive sampling strategy was used to obtain rich descriptions of how individuals experience their swallowing-related problems after surgery. The sampling procedure aimed to establish maximal informant variation by including informants differing in age, gender, marital status, operative level, BMI, diagnosis.

Inclusion criteria were (1) patients older than 18 years after ACSS, (2) normal understanding ability and language expression ability, (3) having undergone ACSS with recurrent laryngeal nerve monitoring,and (4) water-swallowing test grade≥ III. Exclusion criteria were (1) Preexisting vocal cord abnormalities (such as polyps and nodules) and/or any nervous system disease affecting voice or swallowing ability, (2) participation in other research projects, (3) refusal or withdrawal from the interview, and (4) physical or mental disorders. Finally, 22 participants were recruited in the study (Table 1).

**Table 1:** Examples of the analysis with symptoms and psychological experience of dysphagia after ACSS

Quotes	Codes	Sub-categories	Category
…*Although swallowing water is always difficult, I think it is normal after the operation. After all, such a big operation…*	Rationalize the problems	Compromised coping strategies	Coping style
…*I’m having trouble eating now. Is it because your operation has not been done well?… It must be your fault…*	Give vent to the difficulties faced	Negative coping strategies	

### Data collection

Before the semi-structured research interview, the structured interview is used to collect social demographic information, and when the water-swallowing test grade≥ III, it would be included in our interview. The semi-structured interview schedule used in this study was informed by previous literature and agreed upon by all the authors.The semi-structured interview included some prompts to ensure the participants do not deviate from the study’s aim. Interview guides at all time points combined open-ended questions with standardized prompts. However, it was only provided when the participants started discussing irrelevant issues to the study’s aims. The questions covered the patient’s reflection on the experience of related symptoms of dysphagia after ACSS, as well as their psychological characteristics, coping strategies and expectations of others.

The interviewer conducted 1:1 interviews with the participants in a quiet office for 40 to 60 min. Additionally, the schedule was piloted in 2 patients before conducted the data collection. All consenting participants participated in semi-structured interviews at 7 days, 6 weeks, and 6 months after ACSS. The interviewer received 3days of training on qualitative research methods before starting the study, and she is an experienced orthopaedic nurse who has clinical and research interests in the management of patients with cervical spondylosis and was not involved in their clinical care. Collection and analysis of data proceeded simultaneously. The inclusion ended when no new information emerged from additional interviews.

### Symptom identification and assessment

The interview used two different methods to query participants about their symptoms. The first method used open-ended questions to elicit symptoms that came to mind unprompted, such as “What are the symptoms related to your swallowing?“. After the patients had finished answering, they answered according to the symptom list prepared by us, which was formed on the basis of a literature search and expert meeting. The interviewer recorded the frequency of participants’ complaints about each symptom. Results Data were used to measure symptom types and frequency at each time point (7 days, 6 weeks, and 6 months after surgery). This approach allowed us to compare unprompted versus interviewer-prompted symptom complaints, thereby avoiding any potential limitations in the accurate and timely expression of symptoms by the participant.

### Data analysis

Within 24 h after the interview, the researcher transcribed the recording verbatim and documented the emotional and facial changes of the interviewees. When the interview and analysis did not generate new information, data collection was stopped. One researcher converted the recorded content into words, and the other researcher checked the voice and transcriptional interview records to ensure the integrity and accuracy of the content. The process for analysis followed Colaizzi’s seven-stepanalysis [14]: (1) Read the source carefully, (2) Extract phrases or sentences related to the research phenomenon, (3) Encode important statements that occur repeatedly, (4) Categorize the codes and integrate the results, (5) Provide a detailed description of the research phenomenon, (6) Reduce the detailed description to form the structural framework, and (7) Return the research object for verification. Two researchers analyzed the same data separately, and if there were different opinions, the final theme was formed through joint discussion by the research group.The examples of the analysis are in Table 1.

## Results

### Participants

A total of 22 (10 females and 12 males) patients, with years old ranging between 33 and 78 years were interviewed. The clinical characteristics of 22 study participants are shown in Table 2. We followed up on the 22 patients three times. The total interview time for each participant was 58 to 108 min, and the total interview time was 61 h and 52 min. During the whole process, no patient failed to follow up.

**Table 2:** Clinical characteristics of 22 study participants

No.	Gender	Age, years	Marital status	Operative level	BMI	Diagnosis
P1	Male	65	Married	C3/C4,C4/C5, C5/C6	26	CSR
P2	Female	53	Married	C4/C5	25	CSM
P3	Male	48	Married	C4/C5, C5/C6	26	CSM
P4	Female	51	Single	C5/C6	28	CSM
P5	Male	56	Married	C3/C4, C4/C5, C5/C6	25	CSM
P6	Male	62	Married	C4/C5, C5/C6	27	CSM
P7	Female	43	Single	C6/C7	26	CSM
P8	Female	68	Married	C7/T1	24	CSM
P9	Male	33	Single	C3/C4, C4/C5	25	CSM
P10	Female	45	Married	C6/C7	25	CSR
P11	Male	53	Married	C4/C5, C5/C6	28	CSM
P12	Female	50	Married	C5/C6	21	CSM
P13	Male	65	Married	C5/C6, C6/C7	22	GSR
P14	Male	48	Married	C5/C6	24	GSR
P15	Female	46	Married	C4/C5, C5/C6	25	CSM
P16	Female	48	Married	C3/C4,C4/C5, C5/C6	29	CSM
P17	Female	78	Single	C4/C5, C5/C6, C6/C7	25	CSM
P18	Male	72	Married	C4/C5	24	CSM
P19	Female	41	Married	C4/C5	24	CSM
P20	Male	67	Married	C4/C5, C5/C6	21	GSR
P21	Male	42	Married	C5/C6, C6/C7	24	GSR
P22	Male	58	Married	C4/C5, C5/C6	26	GSR

Abbreviations: CSM: cervical spondylotic myelopathy, CSR: Cervical spondylotic radiculopath, C: cervical vertebra, T: thoracic vertebra

### Dominant categories

When analysing the data, the following 3 categories were extracted from the participant interviews: “Subjective symptoms, Coping style and impact on social life”. The 3 categories consist of 10 sub-categories (Table 3).

**Table 3:** Sub-categories and categories

Category	Sub-categories
Subjective symptoms	Physiological symptoms
	Complications
	Negative effects of dysphagia on psychology
Coping with dysphagia	Positive coping strategies
	Negative coping strategies
	Compromised coping strategies
	Wrong coping knowledge
Impact on social life	Restrictions in social life and work
	Changes in intimacy with family
	Lack of professional support from medical staff

### Subjective symptoms

Participants reported various swallowing symptoms after ACSS, and the frequency of complaints varied according to different determination methods: open-ended questions and symptom list prompts. Representative quotes can be found in Table 4.

**Table 4:** Representative quotes from participants describing dysphagia symptoms after ACSS

Sub-categories	Symptom	Time	Quote
Physiological symptoms	Coughing	7 Days	*“I cough as soon as I drink water, but I dare not cough too hard, and I have to control it for fear that the wound will crack.”* (P2)
		6 Weeks	*“These days are much better than before. I can drink without a bottle or straw.”* (P10)
	Swallowing pain	7 Days	*“Swallowing saliva is very painful, not to mention eating, yes,it is like a brush brushing my throat.”* (P3) *“The doctor gave me a small bottle(10ml) of oral liquid for potassium supplement. I had to swallow it three or four times because of the pain.”* (P4)
		6 Weeks	*“Don’t mention it! I feel pain even if I swallow saliva.”* (P2)
		6 Months	*“Now it has been more than half a year since the operation. I often choke while drinking water.”* (P22)
	Difficulty with solids	7 Days	*“It’s very difficult to eat porridge recently, let alone other food. I can’t swallow it. I’m hungry and have no energy all day.”* (P16)
	Food getting stuck	7 Days	*“I ate some mung bean porridge at noon, and the mung bean has been hanging in my throat, which is very uncomfortable.”* (P12)
		6 Weeks	*“I always feel uncomfortable after eating. I have a feeling of obstruction from the throat to the back of the sternum.”* (P11)
		6 Months	*“I dare not eat fish at all, because it is easy to get stuck by fish bones, which is not like this before.”* (P22)
	Increased secretion in throat	7 Days	*“I think my throat has been blocked by phlegm all day, and sometimes I feel it almost overflows from my wound.*” (P8) *“There are many bubble like secretions every day, and a box of such paper (200 sheets) can be used every day.”* (P14)
	Foreign body sensation in throat	7 Days	*“If my cervical vertebra bends forward, this foreign body feeling will be more obvious…The pillow will be uncomfortable if it is high or low.”* (P6)
		6 Months	*“I feel something buried in my throat. But I don’t always have this feeling. It’s paroxysmal.”* (P10)
	Throat burning sensation	7 Days	*“The throat is as dry as burning here. I want to drink water to moisten it, but I can’t drink it.”*(P1) *“When I open my mouth and breathe in the cold air, it will hurt my throat, especially at night.”* (P8)
	The feeling of suffocation	7 Days	*“I dare not even sleep. Once I sleep, I feel suffocated.”*(P8) *“I always feel hard to breathe at night. The air is thin and I can’t breathe.”* (P14)
	Food regurgitation	7 Days	*“I had an orange for lunch. It didn’t take long for the orange juice to flow back from the esophagus to the mouth.”* (P8)
Complications	Lose weight	6 Months	*“I just eat little thing, and I have no strength. I went to weigh myself, and I’ve lost 5 kg in recent months.”* (P15) *“My weight is 4 kg lighter than before, and my hemoglobin is low.”* (P19)
	Sleep disorders	7 Days	*“My throat was dry and painful all night. I need to keep getting up and dropping a few drops of water in my throat. After all, I can’t drink too much at one time.”* (P7)
	Anorexia	6 Months	*“During this period, if I am not very hungry, I will not want to eat.”* (P16)
	Tension before eating	6 Months	*“Once I want to swallow something, I am very nervous because I know I have to suffer again.”* (P2)
Negative effects of dysphagia on psychology	Be anxious about the outcome of the operation	6 Weeks	*“At this time, I was worried whether the reason why I couldn’t swallow anything was because the operation was not done well. I might have to have another operation.”* (P8) *“I have an operation wound on my neck, but I have been coughing all the time. I want to control it, but I can’t control it. This situation will make me recover slowly.”* (P12)
		7 Days	
	Be anxious about self nutrition	6 Weeks	*“What should I do? I took a lot of blood for test before the operation, but now I can’t eat and my nutrition can’t keep up. Now all my pain is in my throat.”* (P5)
	Depressive emotions	6 Weeks	*“I have been very scared these days. I had a friend who couldn’t swallow anything. As a result, he was diagnosed with laryngeal cancer(Whimper)…”* (P4)

When answering open-ended questions, participants complained about swallowing-related questions to varying degrees in three interviews. On the 7th day, the frequency of active complaints about coughing and difficulty with solids was the highest, up to 22 (100%). The next was Swallowing pain and difficulty with liquid, up to 20 (91%). The symptom with relatively few complaints was foreign body sensation, which was only 4 (18%). In addition, anorexia caused by dysphagia was only 2 (9%). Weight loss and anxiety before meals were not mentioned at each time point in the open-ended questions. Some participants reported persistent swallowing problems at the 6-week and 6-month visits. However, it is worth noting that the frequency of reports of burning sensation and food regurgitation decreased to 0. The most common symptom reported by open-ended questions at the 6 weeks and 6 months after ACSS was still difficulty with solids, which was 14 (64%) and 10 (45%) respectively. At 6 weeks and 6 months of assessment, the decrease in swallowing pain was the largest, while the foreign body sensation in the throat increased.

When prompted by the symptom lists, more participants reported swallowing complaints. On the 7th day, some symptoms are more frequent when prompted than opened, such as foreign body sensation in the throat, which was 10 (45%) and 4 (18%) respectively.

### Coping with dysphagia

The second category extracted from the patient interview was the coping strategies initiated by the participants to minimize their swallowing symptoms (Table 5). These strategies were reported at each follow-up time in the open interview section. Strategies were mentioned by 36% (n = 8) of participants 7 days postoperatively, which increased to 82% (n = 18) at 6 weeks and 68% (n = 15) at 6 months.

**Table 5:** Coping strategies reported by study participants

Sub-categories	Symptom	Coping	Quote
Positive coping strategies	Food getting stuck	Humor, Identification, compensate	*“I always get stuck in my throat when I eat. So when I get up and move, I’ll feel much better.”* (P13) *“Eating food with less oil, such as green vegetable leaves, rice and biscuits, is easy to get stuck in the throat. I need to drink water slowly to wash it down.”* (P18) *“For example, if I eat eggs, I will eat steamed egg soup. Compared with boiled eggs, it will not get stuck in my throat.”* (P20)
	Difficulty with solids		*“I choose soft food, chew it slowly, and take smaller bites.”* (P8)
	Throat burning sensation		*“With a plum in my mouth, I will feel much better, or the “throat lozenge” can make me feel comfortable…And yogurt, which makes me feel good”* (P17) *“I learned Chinese medicine. I made some “black wheat and orange drinks”, and the burning feeling disappeared.”* (P17)
	The feeling of suffocation		*“When I feel difficult to breathe, I will sit up for a while or turn over and feel much better.”* (P6)
	Swallowing pain		*“If it is too hot or too cold, it will irritate my throat. I will hold it in my mouth for a while. It will be more comfortable to swallow the water when the temperature is close to my body temperature.”* (P5)
Negative coping strategies		Vent one’s emotions, shrink back	*“I wonder if the operation failed, which completely failed to meet my expectations. I think I may have been worried recently.”* (P20) *“I can’t eat the nutritious food recommended by experts. Because it’s too much trouble, I don’t want to eat.”* (P15) *“I want to stay in the hospital for a few more days, and then leave the hospital after this symptom is completely cured.”* (P9)
Compromised coping strategies		Rationalization, Depression	*“I think it’s normal that I can’t swallow anything after neck surgery. If you don’t ask me, I don’t want to reflect this problem. En… I don’t think it’s a problem.”* (P11)
Wrong coping knowledge		Stereotype	*“I think I have a cold. Once I catch a cold, I will have a lot of phlegm and sore throat, so I take a lot of antibiotics.”* (P8) *“I think I have difficulty breathing because the air in the ward is bad. Last night I went to the corridor to sleep.”* (P14) “I should not eat too oily, too cold food, or hot and dry food like chili and mutton. I dare not eat many things I like, so I don’t want to eat any more.” (P9)

### Impact on social life

The third category extracted from the patient interview was the impact on social life (Table 6). Problems related to swallowing suffering caused them to encounter many obstacles in social life and changed their relationship with their families, and they hoped that medical staff can help them from a professional perspective.

**Table 6:** Impact on the social life of participants and their expectations of others

Sub-categories	Time	Quote
Restrictions in social life and work	6 Weeks	*“Yesterday, when I was on the bus, I felt that there was always phlegm in my throat. I coughed all the time and then put the phlegm in a paper towel. I didn’t want people to look at me. I felt like I had an infectious disease.”* (P9)
	6 Months	*“I used to go to bed at 9:00 pm on time, but now I dare not go to bed because of fear of suffocation, which completely breaks my living habits.” (P8)*
Changes in intimacy with family	6 Weeks	*“I used to live alone. My parents were worried about my situation, so they let me live with them.”* (P7)
	7 Days	*“I’m tired of talking. Recently, my family didn’t call me for fear of affecting my rest, but I really didn’t want to answer the phone.”* (P16)
Lack of professional support from medical staff	7 Days	*“I left the hospital immediately. Although my hands were not numb, I still had difficulty eating. I live far away. What should I do then.”* (P19)
	6 Weeks	*“When I was in hospital, every time I asked the medical staff, they said that it would be better in a few days. Now it has been more than a month, and my problem still hasn’t been solved.”* (P9) *“I hope you can give me some advice on nutrition. After all, I can’t eat much…How to eat is very important for my health.”* (P18)
	6 Months	*“After I left the hospital, I felt abandoned. Except this time when you called me, no one told me how to deal with the problem of swallowing.”* (P17) *“I stayed in the local hospital for eight days after I was discharged from your hospital. I can’t eat. I’m afraid I can’t recuperate well when I go back.”* (P15) *“I have also checked this situation on the Internet, but after all, I don’t believe anything online. I believe what you tell me, so I hope you can give me more information about rehabilitation.”* (P12)

## Discussion

The goal of this study was to better understand the prevalence of postoperative dysphagia symptoms among patients who have undergone ACSS for cervical spondylopathy, directly from the patient’s perspective using rigorous qualitative research methods. We used multiple approaches to ascertain the patient experience related to their postoperative swallow function using both open-ended interviews and interviewer prompts longitudinally over 6 months. Our research results show that there are more symptoms that affect dysphagia after ACSS than previously reported [12–14], and dysphagia can last several months after surgery, even six months after surgery. This requires us to pay long-term attention to such patients. Of course, they also have their own coping strategies, including positive, negative ways. Furthermore, the findings highlight the significance of social limitations, emphasizing the need for increased attention from professionals in addressing this aspect.

Previous studies focused on quantitative research to analyze the risk factors and incidence rate of dysphagia after cervical spine surgery. However, compared with previous studies using cross-sectional or retrospective methods, our study was pre-designed to assess the longitudinal nature of complaints of dysphagia. The study also allowed patients to describe their experiences in their own words and the impact of these symptoms on their quality of life. Our study shows for the first time how patients adapt and self-manage their complaints of dysphagia after anterior cervical surgery.

In our study, early postoperative symptoms of high frequency complaints about dysphagia included solid dysphagia, liquid dysphagia, swallowing pain and cough. The main reason may be due to tissue edema. Miles’ research [15] pointed out that the possible causes of dysphagia in the early stage of ACSS include edema and sensory disturbance caused by local intubation trauma of the upper airway. In addition, the patient’s overall health status is poor, such as the position of the surgical wound, consciousness level, sitting ability, drug effect and fatigue [16]. The structures of the larynx and pharynx, including muscles and nerves, are relatively complex. Surgeons need to move these structures laterally to reach the anterior cervical spine, which can lead to local edema, as well as muscle and neurovascular damage [17, 18]. In the early postoperative period, the burning sensation of the throat is also a symptom that affects dysphagia with more complaints than that in the later period. We inferred that it would be caused by the inflammation of the surgical wound, because at six weeks, no participant mentioned this symptom again. In the later stage, the frequency of food getting stuck, foreign body sensation in the throat is not much different from that of early complaints. This motor and sensory dysphagia may be related to the characteristics of spinal surgery [19]. Spine injury itself, surgery and/or instrument placement, temporary or permanent nerve injury, and soft tissue swelling, which cause swallowing difficulties, are unlikely to recover in the short term []. Muss [21] measured the objective data of 17 patients and found that the impairments in superior hyoid excursion and increased pharyngeal wall thickness are related to dysphagia in the later stage, which would make the patients feel foreign bodies subjectively. Besides, some patients complained about the feeling of suffocation. Although the frequency of this symptom is not much, the consequences will be more serious.

We investigated the subjective symptoms of patients in two ways. Of course, we tried to encourage patients to describe themselves, and the information we got at this time must be the most urgent complaint that patients want to express. From their description, we can also see that they did not realize the importance of this problem. Most patients did not stay in hospital for long. The surgery solved their previous symptoms, so they did not want to mention the second swallowing problem, believing it was temporary. Helldén also found [22] this problem after thyroid surgery, which also led medical workers to be unaware of this problem. Subjective dysphagia is usually minimized by medical workers, because these patients are considered “safe” and have no risk of aspiration. However, these complaints should not be ignored, as our research findings clearly demonstrate that subjective dysphagia have a significant impact on patients’ quality of life. Subjective dysphagia is very troublesome for patients, and may be exacerbated by the lack of counseling or advice strategies to help reduce the impact of these symptoms on daily life [23, 24].

Patients also had their own ways of coping with such problems, including positive, negative, compromise, and of course, wrong ways to deal with them. Positive coping strategies indicate their desire to change this symptom. But our data also showed that there were still some patients who continue to struggle with dysphagia in the later stage without the guidance of health care professionals. The methods were not necessarily right, and they need professional guidance urgently. We need to continue to pay attention and develop rehabilitation treatment methods for this unfortunate group. What our findings emphasize is that healthcare professionals should pay more attention to patients who adopt negative and compromise strategies, which will bring them adverse consequences [25]. Higuchi’s research shows that negative coping strategies of spinal surgery patients negatively regulate the relationship between pain and physical, psychological and social health [26]. Of course, the duration and experience of symptoms will also affect the coping style of patients. Angelini’s research [27] also mentioned this problem, and healthcare professionals need to provide personalized measures.

The participants’ complaints also mentioned the impact of dysphagia on their life, work and family. After the operation, they move towards life and inevitably depend on others. They can only rely on the care and support of family members and friends, sometimes even elderly parents. This is a common problem for patients after surgery. This dependence on others is usually related to fear, depression and insecurity [28]. Indeed, the changes in social life and the psychological problems mentioned in the subjective symptoms section interact, because they are faced alone most of the time without the guidance of professionals [29], which also suggests that we need to establish a continuous rehabilitation program to solve this problem. Of course, the family participation model [30] can help family members better participate in the care of patients, understand the emotional cycle of patients and give encouragement, actively communicate with patients, increase intimacy, and improve the psychological resilience of patients, so as to better provide effective support to patients.

### Limitations

There are several limitations to our study. First, we only followed up with each patient for six months after surgery, so there is no information about long-term follow-up. Second, In the early interview, the patient had relatively serious dysphagia, with a lot of secretions in the throat, and was unwilling to speak more. Only structural interviews were used to encourage the patient to communicate more about their postoperative feelings, or family members were involved in the communication, which may lead to the patient not fully describing their experiences. However, it may be interesting for family members to participate in the interview, which can provide information about the direct or indirect impact of dysphagia, as well as the impact of dysphagia on their common life. Third, This study only considered the feelings of patients with dysphagia assessed by water-swallowing test. In fact, many patients after ACSS have problems related to swallowing, which have troubled their life. These patients were not included in the study.

## Conclusions

In conclusion, qualitative research methods can be used to better understand the personal experience of dysphagia after ACSS. The rehabilitation process of dysphagia is a complex process involving the interaction of many physical, emotional and social factors. Moreover, dysphagia after cervical surgery has unique characteristics, which require special evaluation methods to better screen the problems of dysphagia after surgery. Raising the awareness of healthcare professionals about these complexities will help to provide high-quality and evidence-based care and ensure that service delivery is truly patient-centred.

Our study also shows that patients with dysphagia after ACSS lack support from healthcare professionals. They feel abandoned and adjust their strategies without support and guidance. Therefore, healthcare professionals should do a better job in providing health care support in the early or late postoperative period to ensure the improvement of health outcomes and patients’ quality of life.

## Data Availability

The datasets during and/or analysed during the current study available from the corresponding author on reasonable request.
